# Analysis of Lip Pressure and Quality of Life Following Gummy Smile Treatment

**DOI:** 10.34172/joddd.44115

**Published:** 2026-03-30

**Authors:** Paula Amaral Salgado Poliseli, Marcelo Palinkas, Nicole Barbosa Bettiol, Bianka Jurca Gonçalves da Motta, Paula Napolitano Gonçalves, Pedro Bastos Cruvinel, Selma Siéssere, Simone Cecilio Hallak Regalo

**Affiliations:** ^1^Department of Basic and Oral Biology, Ribeirão Preto School of Dentistry, University of São Paulo, Ribeirao Preto, Brazil; ^2^National Institute of Science and Technology Translational Medicine (INCT-TM), Porto Alegre, Brazil

**Keywords:** Botulinum toxin, Glasgow benefit inventory, Gummy smile, Hyaluronic acid, Lips

## Abstract

**Introduction:**

A gummy smile is an aesthetic condition that can negatively impact self-esteem and quality of life. This longitudinal study aimed to evaluate the effects of gummy smile treatments with botulinum toxin type A and/or hyaluronic acid on lip pressure and the perceived quality of life in adult individuals.

**Methods:**

Thirty adult individuals aged 18‒65 years, with>2 mm of gingival exposure when smiling, participated in the study. They were randomly assigned to three groups: botulinum toxin (n=10), hyaluronic acid (n=10), and a combination of both (n=10). Lip pressure was measured using the Biofeedback Pró-Fono device at baseline and 15, 30, 90, and 180 days after the procedures. Quality of life was assessed using the Glasgow Benefit Inventory, which was applied 15 days after treatment. Statistical analyses included repeated-measures ANOVA with Bonferroni correction and t-tests (*P* ± 0.05).

**Results:**

All the groups showed variations in lip pressure over time, with a gradual increase up to 180 days and statistically significant differences. Intergroup comparisons across time intervals were not assessed. All three groups showed improvements in the quality of life, with the combined treatment standing out.

**Conclusion:**

Aesthetic treatments for gummy smile resulted in significant changes in lip pressure over time and were associated with improvements in the quality of life.

## Graphical Abstract

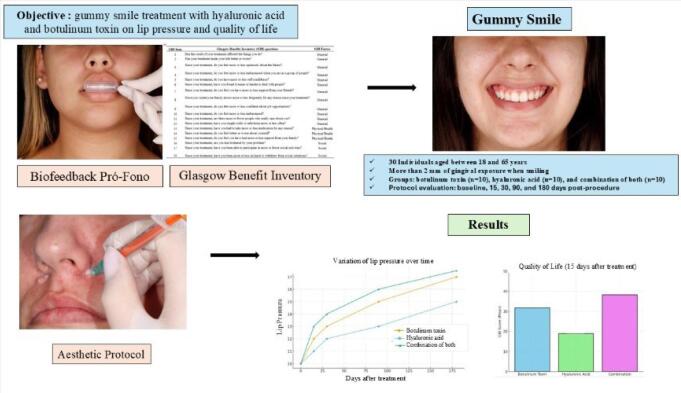

## Introduction

 Smile aesthetics gos beyond tooth color and alignment and is also influenced by the exposure and symmetry of the gums in relation to the teeth and lips.^1^ A harmonious smile features minimal upper gingival exposure, alignment between the gingival line and the upper lip, the presence of interdental papillae, teeth with proper anatomy, and a lower lip that runs parallel to the incisal edge of the anterior teeth.^2^ In this context, the smile plays a crucial role in facial expression and in the perception of self-esteem.^3^

 A gummy smile is a common aesthetic condition characterized by the exposure of > 3 mm of gingival tissue when smiling.^4^ It has a multifactorial cause, including skeletal, dental, or muscular alterations such as hyperactivity of the upper lip, and is often associated with dissatisfaction with appearance.^5^ Treatment should be individualized according to the etiology, ranging from surgeries to minimally invasive approaches such as myomodulation with botulinum toxin type A and hyaluronic acid.^6,7^

 Botulinum toxin type A, derived from the bacterium *Clostridium botulinum*, works by inhibiting the release of acetylcholine at neuromuscular junctions, promoting temporary muscle paralysis.^8^ Its application, especially in the facial muscles, helps reduce gingival exposure by controlling their hyperactivity.^9^ Hyaluronic acid, a polysaccharide naturally present in the body, has widespread use in aesthetic procedures due to its properties of volumizing, hydration, and tissue support.^10^

 Both treatments offer advantages such as reversibility, safety, and quick aesthetic results. However, possible complications include asymmetries, lip ptosis (in the case of botulinum toxin), and, more rarely, tissue necrosis due to compression or embolization.^11^ The success of the interventions depends on detailed knowledge of facial anatomy and physiology, especially the muscular function in the perioral region.^12^

 Despite the increasing use of botulinum toxin and hyaluronic acid in aesthetic dentistry, clinical studies systematically investigating their isolated and combined effects on gummy smile, particularly regarding muscle function and psychosocial well-being, are still scarce. In this context, it becomes essential to understand not only the aesthetic efficacy of these techniques but also their functional and subjective impacts on patients’ lives.

 Therefore, this observational study aimed to evaluate the effects of gummy smile treatments with botulinum toxin type A and/or hyaluronic acid on lip pressure and perceived quality of life in adult individuals. The null hypothesis of this study was that there would be no significant differences in lip pressure and perceived quality of life following the proposed treatments for gummy smile.

## Methods

###  Sample

 This longitudinal study was approved by the Ethics Committee of the School of Dentistry of Ribeirão Preto, University of São Paulo, Brazil (protocol #76818423.3.0000.5419). All the participants signed an informed consent form.

 To determine the required sample size for the study, G*Power 3.1.9.2 software (Franz Faul, University of Kiel, Germany) was used, with a significance level of α = 0.05. An effect size of 1.26 and a test power of 84% were established based on electromyographic activity of the upper segment of the orbicularis oris muscle. These parameters were defined from data obtained in a pilot study conducted by the same research group, involving five individuals.^13^ Thus, it was established that each treatment group should have at least 10 individuals.

 Sample selection was conducted at a specialized dental clinic, where a previously calibrated orthodontist performed a clinical evaluation to identify complaints of gummy smile. The evaluation included medical and dental anamnesis, facial proportion measurements using a Willis compass (between the interpupillary line and the labial commissure at rest, and between the base of the nose and the chin at maximum dental occlusion), and the distance from the gingival zenith of the maxillary central incisor to the lower edge of the upper lip, measured with a millimeter ruler. These measurements aided in the initial characterization of the gummy smile. Subsequently, a digital caliper was used to measure the distance between the stomion point of the upper lip and the base of the anterior nasal spine.

**Figure 1:**
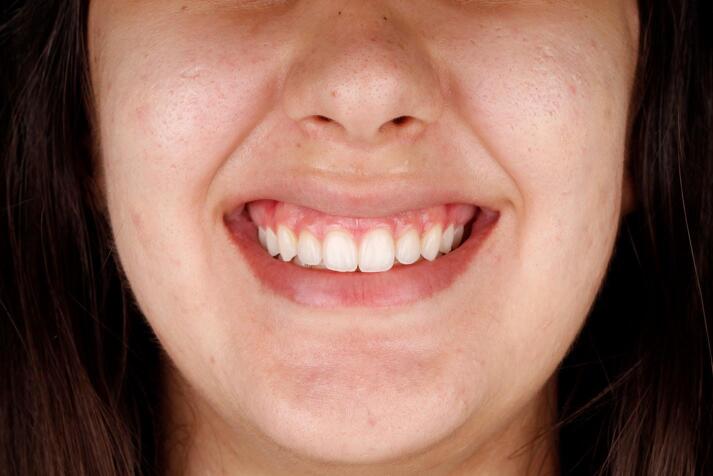
Gummy smile

 The study included individuals of both sexes, aged 18‒65 years, who presented with > 2 mm of gingival exposure when smiling (Figure 1) and lip length within normal ranges (18–24 mm for men and 16–20 mm for women), with no absolute contraindications to the use of botulinum toxin or hyaluronic acid. Exclusion criteria included individuals with ± 2 mm of gingival exposure, lip length below the normal ranges, increased vertical maxillary growth, mouth breathing, history of maxillofacial surgeries or lip lesions, motor or neurological disorders, presence or history of cleft lip and previous treatments with botulinum toxin or hyaluronic acid in the nasal or labial region within the past six months.

 The observation and analysis were conducted by an independent evaluator who had no prior knowledge of the procedure performed by the lead surgeon in order to minimize bias.

###  Gummy Smile Treatment

**Figure 2:**
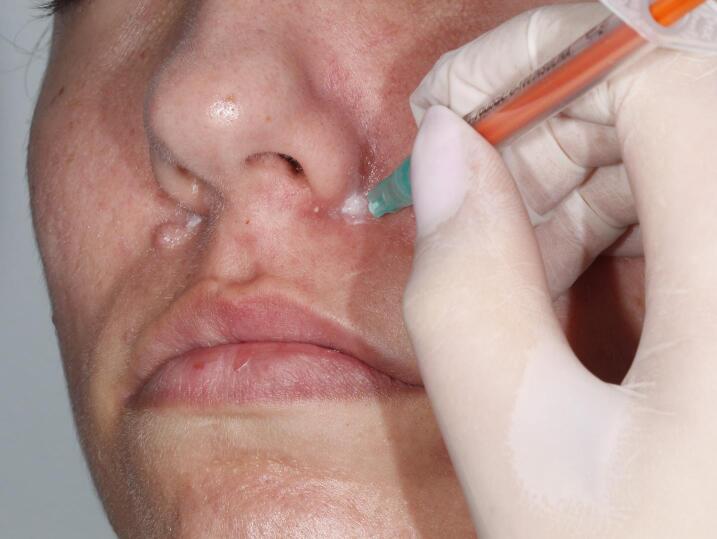
Aesthetic protocol

 For the application of botulinum toxin type A, a vial containing 100 units (Nabota®, Daewoong Pharmaceutical) was reconstituted with 2 mL of sterile saline solution, using two 1-mL insulin syringes with 26-G needles. The solution was then aspirated in a volume of 4 units (0.08 mL) into a similar syringe, with the needle replaced by a 32 × 4-mm needle. The toxin was injected at a depth of 4 mm, with 2 units (0.04 mL) per injection in the region of the anterior nasal spine.^14^ In cases treated with hyaluronic acid (Rennova® Ultra Deep, Panaxia LTD Bat Sheva 1, Israel), 0.2 mL of the substance was applied to the anterior nasal spine using Rennova® syringes and needles to ensure precision and safety.^10^ All the procedures were performed by a single dental surgeon specialized in orofacial harmonization, in a specialized dental clinic located in Ribeirão Preto, São Paulo, Brazil. Post-procedure instructions and care were provided to all patients after the applications (Figure 2).

###  Orofacial Tissue Pressure Analysis

**Figure 3:**
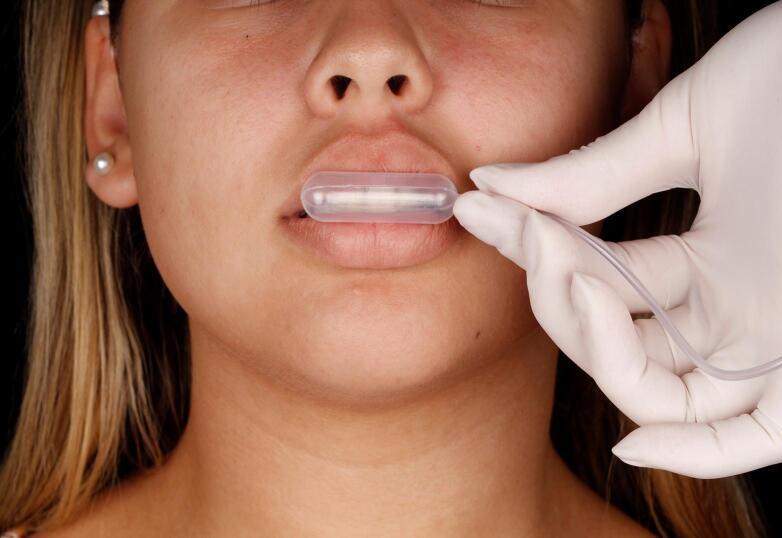
Lip pressure assessment

 Individuals underwent lip pressure measurement, expressed in kilopascals (kPa), using the Biofeedback Pró-Fono device – Lip and Tongue Pressure Measurement (PLL Pró-Fono), an instrument validated by the Brazilian National Health Surveillance Agency (Anvisa). The device consists of a pressure sensor connected to an electronic board, both housed in a plastic casing. The sensor is connected to a flexible plastic tube, which is attached to a device with an air bulb.^15^ During the assessment, the individual remained seated with a back support, while the air bulb was positioned transversely between the lips (Figure 3). They were then instructed to exert maximum lip pressure on the bulb for 4 seconds, followed by a 30-second rest interval. The procedure was performed three times. The recorded lip pressures were compiled, and the average of the obtained values was calculated using specialized software (PLL Pró-Fono).

###  Quality of Life Analysis

 The instrument used to measure quality of life was the Glasgow Benefit Inventory.^16,17^ The participants answered the questionnaire based on their personal experiences and perceptions, using a 5-point Likert scale ranging from ‘much worse’ to ‘much better’ or from ‘very dissatisfied’ to ‘very satisfied.’

 The Glasgow Benefit Inventory was administered 15 days after the gummy smile treatment. The questionnaire included 12 items related to general changes in health status, covering psychosocial aspects classified under the ‘general’ category. Three additional questions addressed physical health, focusing on factors such as medication use and frequency of medical or dental visits after completion. The final three questions concerned the level of social support needed due to the evaluated condition.

 The overall Glasgow Benefit Inventory score was calculated from the responses to assess the impact of the aesthetic intervention on quality of life. Scores assigned to questions in each subscale (General, Physical/Health, and Social) were summed and divided by the number of corresponding items: 12 questions for the General Well-Being subscale, 3 for Physical/Health Well-being, and 3 for Social Well-being. The arithmetic mean of the three subscales resulted in the final Glasgow Benefit Inventory score, ranging from -100 to + 100. Positive values indicate perceived improvement in the quality of life following lip treatment, while negative values suggest deterioration. A score of zero represents no perceived change in the quality of life.

###  Statistical Analysis

 After data collection, the Shapiro-Wilk normality test was performed, indicating a normal distribution. Statistical analysis was conducted using SPSS 20.0 (SPSS Inc., Chicago, IL, USA). Repeated-measures ANOVA with Bonferroni correction and t-tests were used, adopting a significance level of 5% (*P* ± 0.05). Additionally, descriptive frequency analysis of the Glasgow Benefit Inventory data was performed, including means, standard deviations, medians, and quartiles (25‒75%).

## Results

 Gingival display analysis demonstrated that all treatments were effective in reducing gingival exposure over time. In the botulinum toxin group, the mean gingival display decreased from 4.09 mm at baseline to 2.20 mm at 15 days, representing a mean reduction of 1.89 mm, followed by a gradual increase at subsequent time intervals. In the hyaluronic acid group, the mean gingival display decreased from 5.25 mm to 2.95 mm at 15 days (a mean reduction of 2.30 mm), with greater variability and a tendency to return toward baseline values at 180 days. In the combined treatment group, the greatest reduction was observed, with mean values decreasing from 4.75 mm at baseline to 1.95 mm at 15 days (a mean reduction of 2.80 mm) and maintaining more stable results over time compared to the other groups. No inferential statistical comparisons or correlation analyses were performed between gingival display reduction and other evaluated outcomes.

Table 1 shows the differences in mean lip pressure values at baseline and after gummy smile treatment with botulinum toxin type A, hyaluronic acid, and their combination. In all cases, a significant difference was observed in the progression of the means over time. Intergroup comparisons across time intervals were not assessed. For the treatment with botulinum toxin type A, a significant difference was identified (*P* = 0.03), with an increase in lip pressure at 15 days, followed by a reduction at 30 days and gradual growth maintained between 90 and 180 days. Similarly, the treatment with hyaluronic acid also showed a significant difference (*P* = 0.03), with an increase in pressure at 15 days, a decrease at 30 days, and subsequent progressive increase between 90 and 180 days. Meanwhile, the combined treatment with botulinum toxin type A and hyaluronic acid demonstrated a significant difference (*P* = 0.00), with a reduction in lip pressure at 15 days, followed by a gradual increase from 30 days onward, continuing to rise up to 180 days.

**Table 1:** Differences in mean lip pressure values ( ± standard error) at baseline and after gummy smile treatment

**Gummy smile treatment**	**Periods**						
	**Baseline**	**15 days**	**30 days**	**90 days**	**180 days**	***P*** **-value (ANOVA)**	***P*** **-value (Periods vs. Periods)**
Botulinum toxin	11.4 ± 1.6	14.1 ± 2.7	13.3 ± 1.9	15.5 ± 3.1	17.6 ± 2.7	0.03	-
Hyaluronic acid	10.8 ± 1.4	12.8 ± 2.4	12.1 ± 2.3	13.3 ± 2.7	13.8 ± 2.2	0.03	-
Both	9.3 ± 0.4	9.1 ± 0.3	9.3 ± 0.3	10.1 ± 0.3	11.8 ± 0.3	0.00	0.04 (II vs.V)

vs., versus. Significant differences measured by repeated-measures ANOVA with Bonferroni correction (*P* ± 0.05).

Table 2 presents the results of the Glasgow Benefit Inventory 15 days after gummy smile treatment with botulinum toxin type A, hyaluronic acid, and their combination. All the groups showed improvements in the quality of life, reflected by increased means and medians in the domains of General Health, Physical/Health, and Social. However, the magnitude of this improvement varied between treatments, both in total score and in the analyzed subdomains, with emphasis on the combined treatment.

**Table 2:** Differences in Glasgow Benefit Inventory scores with respective subdomains after 15 days of gummy smile treatments

**Treatments**	**GBI**	**Values**	
Botulinum toxin	Total GBI	Mean (SD)	+ 31.8 (20.24)
		Median (25‒75%)	+ 26.28 (19.44‒45.48)
	GBI subdomains		
	General	Mean (SD)	+ 30.41 (22.22)
		Median (25‒75%)	+ 29.16 (13.53‒42.70)
	Physical/Health	Mean (SD)	+ 19.99 (15.31)
		Median (25‒75%)	+ 16.66 (12.49‒33.33)
	Social	Mean (SD)	+ 49.99 (29.44)
		Median (25‒75%)	+ 33.33 (33.33‒70.82)
Hyaluronic acid	Total GBI	Mean (SD)	+ 18.88 (15.97)
		Median (25‒75%)	+ 14.58 (10.41‒28.81)
	GBI subdomains		
	General	Mean (SD)	+ 16.66 (15.21)
		Median (25‒75%)	+ 10.41(6.24‒30.20)
	Physical/Health	Mean (SD)	+ 16.66 (11.11)
		Median (25‒75%)	+ 16.66 (12.49‒20.82)
	Social	Mean (SD)	+ 23.33 (26.29)
		Median (25‒75%)	+ 16.66 (0‒37.49)
Both	Total GBI	Mean (SD)	+ 38.29 (24.43)
		Median (25‒75%)	+ 34.72 (22.56‒59.02)
	GBI Subdomains		
	General	Mean (SD)	+ 43.32 (26.22)
		Median (25‒75%)	+ 39.58 (26.03‒64.58)
	Physical/Health	Mean (SD)	+ 26.66 (16.10)
		Median (25‒75%)	+ 16.6 (12.49‒33.33)
	Social	Mean (SD)	+ 44.99 (36.04)
		Median (25‒75%)	+ 33.33 (16.66‒74.99)

SD, standard deviation; GBI, Glasgow Benefits Inventory mean, standard deviation, median, and quartile values (25‒75%).

## Discussion

 The null hypothesis of this study assumed that there would be no significant differences in lip pressure or in individuals’ perception of the quality of life after the application of botulinum toxin type A, hyaluronic acid, or their combination. However, the findings demonstrated differences in these parameters over time, indicating that the studied interventions produced measurable effects on lip pressure and quality of life in individuals treated for gummy smile. Intergroup comparisons across time intervals were not assessed, which limits direct comparisons between treatments over time.

 Treatment of gummy smile with hyaluronic acid demonstrated significant changes in lip pressure over time. After the application of hyaluronic acid, an increase in pressure was observed at 15 days, followed by a decrease at 30 days. From that point onward, there was a gradual increase in lip pressure between 90 and 180 days, with values at the end of this period surpassing those recorded before the procedure, showing statistical significance. This behavior may align with the concept of functional myomodulation, whereby hyaluronic acid filler provides not only a volumizing effect but also structural support and muscle rebalancing, thus modulating muscle force vectors.^18^ Although originally applied to other areas of the face, the concept was demonstrated here in the modulation of the musculature associated with the gummy smile.

 The literature supports these findings by highlighting the role of hyaluronic acid as a myofunctional support agent. Studies indicate that its application in strategic areas of the midface can directly influence the action of muscles such as the levator labii superioris, the alar part of the nose, and the zygomaticus minor, all involved in the excessive gingival display during smiling.^5^ Based on this, it is plausible to attribute the improvement in lip pressure to the stabilizing effect of the filler, which provides mechanical resistance to muscle traction, promoting a more favorable functional balance.

 The use of hyaluronic acid in regulating the dynamics of the upper lip and reducing gingival display has proven effective, especially due to its reversible and aesthetic effects.^19^ Complementing this approach, studies have shown that bolus injections of hyaluronic acid directly over the periosteum of the piriform fossa and the anterior nasal spine, in volumes between 0.2 to 0.3 mL, promote significant reductions in gingival display.^10^ This technique acts specifically on the levator labii superioris muscle, limiting its range of traction, reducing the elevation of the lip during smiling, which is in accordance with the concept of functional myomodulation.^20^

 Thus, the results of this study also confirm changes in lip pressure after the application of hyaluronic acid, indicating a direct effect not only on the elevator muscles but also on related musculature, considering their insertion in the skin and mucosa of the upper lip. Furthermore, the temporal pattern of pressure increase after 15 days suggests that the filled tissue may be undergoing a period of biomechanical accommodation, followed by progressive functional adaptation, a hypothesis that warrants further investigation in future studies with more detailed histological analysis.

 In the treatment with botulinum toxin type A, a similar functional pattern was observed. After application to the elevator muscles of the upper lip, there was an initial increase in lip pressure at 15 days, followed by a decrease at 30 days, and then a gradual increase between 90 and 180 days. By the end of this period, the pressure was higher than that recorded before the procedure. Botulinum toxin initially reduces the contraction strength of the target muscles, with gradual recovery after 90 days as the neuromuscular blockade reverses.^21,22^

 This effect is also described, with maximum reduction of muscle activity occurring between two and four weeks after application, an average duration of 12 weeks, and a gradual return to baseline condition in about 24 weeks.^23^ It is believed that the initial increase in lip pressure at 15 days may be attributed to a compensatory adjustment of the muscles adjacent to those directly affected by the botulinum toxin. This hypothesis is supported by electromyographic studies of facial muscles, which demonstrate a redistribution of muscle tone as a functional response to the aesthetic treatment.^24,25^ Considering the anatomy of the levator labii superioris muscle and its insertion in the skin and mucosa, the correlation between the strength of these muscles and lip pressure becomes evident. The findings of this study, which indicate significant changes in lip pressure over time, reinforce the notion of muscle neuromodulation promoted by botulinum toxin.^26^

 When analyzing the combination of hyaluronic acid and botulinum toxin, a slight reduction in lip pressure was observed at 15 days, followed by a progressive increase at 30, 90, and 180 days. Although there are few clinical studies on this combination, some authors propose a conceptual approach that integrates the volumetric and structural effects of hyaluronic acid with the neuromuscular blockade promoted by botulinum toxin, aiming to optimize functional outcomes,^14^ although without specifying the anatomical points or injection volumes used.

 This therapeutic combination has also been described as an emerging strategy for the treatment of gummy smile, suggesting that hyaluronic acid would limit muscle movement and prevent full contraction, thereby restricting lip elevation during smiling, while botulinum toxin would provide selective relaxation.^27^ Such synergy may explain the gradual modulation of lip pressure observed in this study. The combined treatment appears to provide both neuromodulatory and structural support, which may explain the greater stability observed over time compared to isolated approaches.

 The results obtained through the Glasgow Benefit Inventory demonstrated that all treatments evaluated for gummy smile (botulinum toxin, hyaluronic acid, and their combination) promoted a perceived improvement in the quality of life after 180 days. However, it was observed that the intensity of this improvement varied among the groups, both in the overall Glasgow Benefit Inventory score and in the subdomains of General Health, Physical/Health, and Social.

 The group treated with botulinum toxin alone exhibited a total mean Glasgow Benefit Inventory score of + 31.8 (standard deviation 20.24), with a median of + 26.28 (19.44–45.48). Among the subdomains, the highest average score was observed in the Social domain ( + 49.99; standard deviation 29.44), indicating that individuals perceived a considerable social impact after treatment, possibly related to aesthetic improvement and increased self-confidence in interpersonal interactions. These findings are consistent with the literature, which highlights botulinum toxin as a significant promoter of improved quality of life and psychosocial well-being, especially when used in facial aesthetic procedures.^28^

 The group treated with hyaluronic acid alone also showed improvements in the Glasgow Benefit Inventory scores, but to a lower extent. The overall GBI mean score was + 18.88 (standard deviation: 15.97), with a median of + 14.58 (10.41–28.81). Despite the positive gains, these values were noticeably lower than those observed with botulinum toxin. The Social subdomain was again the most impacted ( + 23.33; standard deviation: 26.29), although with a lower average compared to the other groups. This suggests that while hyaluronic acid had beneficial effects, its subjective perception in terms of social benefit may be more limited.

 The results indicated that facial harmonization influenced the gummy smile not only from an aesthetic perspective but also in functional and psychosocial aspects. Previous studies on the use of hyaluronic acid for lip augmentation have shown similar benefits, as both procedures involve modulation of the perioral musculature, positively impacting self-perception, confidence, and the quality of social interactions.^17^ These findings suggest that functional changes in lip pressure may be associated with improvements in gingival display, although this relationship was not formally tested.

 On the other hand, the best results were observed in the group that received a combination of botulinum toxin and hyaluronic acid. The overall mean score on the Glasgow Benefit Inventory was + 38.29 (standard deviation 24.43), with a median of + 34.72 (22.56–59.02). This group achieved the highest average scores across all subdomains, especially in General Health ( + 43.32; standard deviation 26.22) and Social ( + 44.99; standard deviation: 36.94). This pattern supports the hypothesis that the combined approach could enhance the individual benefits of each technique, promoting a more comprehensive and satisfying experience for the patient.

 Positive scores in the subscales of the Glasgow Benefit Inventory confirm that the aesthetic intervention yielded favorable impacts beyond appearance, also affecting psychosocial and functional aspects of the individuals’ lives. These findings align with research highlighting the positive effects of minimally invasive aesthetic procedures on self-esteem, body image, and interpersonal relationships.^29,30^ Improvement in self-image perception, especially in such a visible area as the face, may have triggered a positive feedback loop in social interactions, resulting in subjective gains in well-being and personal confidence.

 Despite the clinical relevance of the findings, this study had some limitations. Although the follow-up period was adequate to assess temporary effects, it may not fully capture long-term variations, especially considering the natural biodegradation of hyaluronic acid and the reversibility of botulinum toxin effects. Studies with extended follow-up periods could help validate and expand these findings, providing a more comprehensive understanding of the efficacy and durability of treatments for gummy smile. Additionally, gingival display reduction was quantified over time, indicating that all treatments promoted a reduction in gingival display, with greater reduction and stability observed in the combined treatment group. However, no correlation analysis was performed between the reduction in gingival display and lip pressure or quality of life outcomes, which limits the understanding of the relationship between aesthetic improvements and functional or patient-reported outcomes. Future studies should explore these associations to better elucidate the interaction between these variables.

## Conclusion

 The findings indicated that minimally invasive treatments for gummy smile using botulinum toxin type A and/or hyaluronic acid promoted time-dependent changes in lip pressure, with improvements in the quality of life.

## Competing Interests

 The authors declare no conflicts of interest.

## Ethical Approval

 The present longitudinal study was approved by the Ethics Committee of the School of Dentistry of Ribeirão Preto, University of São Paulo, Brazil (protocol #76818423.3.0000.5419).
